# Outcomes of Adjuvant Therapy for Stage IA Serous Endometrial Cancer

**DOI:** 10.7759/cureus.3387

**Published:** 2018-09-29

**Authors:** Elysia Donovan, Clare J Reade, Lua R Eiriksson, Gregory R Pond, Nikita Arora, Lorraine Elit, Sadaf Memon, Sachi Voruganti, Maltibehn Patel, Waldo Jimenez, Mazurka John, Iwa Kong

**Affiliations:** 1 Radiation Oncology, Juravinski Cancer Centre-McMaster University, Hamilton, CAN; 2 Obstetrics and Gynecology, Juravinski Cancer Centre-McMaster University, Hamilton, CAN; 3 Epidemiology and Public Health, Juravinski Cancer Centre-McMaster University, Hamilton, CAN; 4 Miscellaneous, Juravinski Cancer Centre-McMaster University, Hamilton, CAN; 5 Pathology, Juravinski Cancer Centre-McMaster University, Hamilton, CAN

**Keywords:** serous, endometrial, cancer, early-stage, adjuvant

## Abstract

Purpose: Serous adenocarcinoma is a rare, aggressive histologic subtype of endometrial cancer with a high rate of recurrence and a poor prognosis. The optimal adjuvant treatment for early-stage patients is unclear. Our objective was to evaluate the outcomes of stage IA serous endometrial cancers only treated at a single institution and determine whether our current approach of chemotherapy plus vaginal brachytherapy (VBT) is sufficient.

Methods: A retrospective chart review of our institution's pathology database, including all cases of stage IA serous endometrial carcinoma from 2000-2014 was completed. Kaplan-Meier estimates were calculated for Overall and Recurrence-Free Survival (OS and RFS); hazard ratios were calculated using Cox proportional hazards modeling for independent prognostic factors.

Results: There were 63 patients with stage IA serous endometrial cancer of whom 79.4% were surgically staged. Percent RFS was 76.5% at five years while OS was 84.7% for the whole cohort. One of the 23 patients receiving VBT and chemotherapy recurred at the vagina versus four of 32 patients who were observed. Two patients in the observation group recurred in the pelvis while there were no first pelvic recurrences in the VBT and chemotherapy group (non- significant). Overall survival was 95% in the brachytherapy and chemotherapy group versus 79.6% in the observation group (non-significant). Post-operative management included observation (n=33), combination VBT and chemotherapy (n=21), or chemotherapy with or without external beam radiation therapy (EBRT) (n=9).

Discussion: We report one of the largest cohorts of serous endometrial cancer stage IA patients. Our results emphasize the inferior RFS and OS of stage IA serous versus endometrioid endometrial cancer patients. While some centers continue to use EBRT for these patients, our results demonstrate low pelvic recurrence rates with radiotherapy limited to VBT, as well as the high systemic risk regardless of treatment. We advocate for combination chemotherapy and brachytherapy given the poor outcomes in these patients.

## Introduction

Serous adenocarcinoma is a rare subtype of endometrial cancer that behaves aggressively, with a high risk of both local and distant recurrence. While the serous subtype represents less than 10% of all uterine cancers, it accounts for almost 50% of relapses and deaths for this disease (1). A recent Gynecologic Cancer InterGroup review suggests more than half of serous endometrial cancer patients present with stage I disease [1]; however, overall survival (OS) rates for early stage serous endometrial cancer have been reported as low as 65%-85% [2-5]. The definitive treatment for serous endometrial cancer is hysterectomy with bilateral salpingo-oophorectomy and surgical staging [2,4], which typically includes omentectomy and pelvic and para-aortic lymphadenectomy. Patients are often upstaged when surgical staging is performed, and studies have suggested a survival benefit with pelvic lymphadenectomy [6].

The increased risk of recurrence and the lower overall survival (OS) rate warrants a consideration of adjuvant therapy for patients with an early-stage disease, however, inconsistencies in the treatment and outcome reported in the literature has led to a lack of consensus on an optimal regimen. The benefit of adjuvant therapy for patients with stage IA disease is less certain than for patients with a more advanced stage disease. Observation has been reported as sufficient in some cases [7-10] while other studies defend the use of pelvic radiotherapy or vaginal brachytherapy (VBT) to obtain local control [8,11] and chemotherapy, given the high risk of distant metastasis [12-13]. Balancing sufficient treatment with an avoidance of potential toxicities [14-15] is therefore challenging and requires further investigation. The uncommon nature of this entity, as well as the reluctance of centers to randomize patients to treatments other than chemotherapy and radiotherapy, has, so far, precluded the performance of randomized trials.

At our institution, the majority of patients were observed or given adjuvant therapy based on physician discretion until the year 2009 when a regimen of adjuvant carboplatinum-based chemotherapy and VBT was introduced. Our study aims to describe the outcomes of all stage IA serous patients treated at our center over the past 15 years and compare patients who received adjuvant chemotherapy and VBT with those that had no adjuvant therapy.

## Materials and methods

A retrospective chart review inclusive of all cases of “serous carcinoma” of the endometrium from 2000 to 2014 was completed. This project was reviewed and approved by the local ethics board. All reports of “serous carcinoma” in our central pathology database were identified and reviewed to confirm uterine disease site and stage. A central review at our center was done in all cases, and a gynecologic pathologist reviewed all pathology reports for the purpose of this study. Patients with stage IA ( International Federation of Gynecology and Obstetrics (FIGO) 2009 staging) pure or mixed serous histology (minimum 5% serous) who underwent total hysterectomy and bilateral salpingo-oophorectomy with or without surgical staging were included. Patients with a carcinosarcoma component to their histology were excluded.

Adjuvant therapies were recorded for each patient and included vaginal brachytherapy (VBT), pelvic external beam chemotherapy (EBRT), chemotherapy, or observation.

VBT was delivered using a vaginal cylinder for a total dose of 3150 cGy in three fractions once weekly, prescribed to the vaginal surface of the upper half of the vagina. Adjuvant EBRT was delivered using a four-field technique for a dose of 4500 cGy in 25 fractions. Chemotherapy included single agent carboplatin ( area under the curve (AUC) 5-6) or a combination of carboplatin (AUC 5-6) and paclitaxel (175 mg/m2 over three hours) every three weeks for six cycles. Those who received no adjuvant therapy were followed by physical examinations at intervals of three to six months for three years, then annually until five years. Patients were censored at the last recorded visit at the cancer center. Date of recurrence or date of death and duration of follow-up were used to calculate recurrence-free survival (RFS) and OS in months. The site of recurrence was recorded as local in the vagina, in the pelvis, or distant disease.

Summary statistics were used to describe the patient characteristics, pathology, treatment characteristics, and outcomes. The Kaplan-Meier method was used to calculate OS and RFS outcomes. Cox proportional hazards regression was used to investigate factors prognostic for OS and RFS. Stepwise selection was used to attempt to construct an optimal model of prognosticators for outcomes of interest.

A subgroup analysis was performed, including only those patients who were surgically staged. An analysis was performed comparing outcomes between the largest groups of patients, those who received VBT plus chemotherapy, and those who received no adjuvant therapy. Fisher’s exact test was used to compare rates of recurrence (any versus none) while the log-rank test was used to compare OS and RFS outcomes between these groups. Statistical significance was defined as a p-value<0.05 and all tests were two-sided. Statistical analysis was performed using SAS Version 9.2 software (SAS Institute, North Carolina, US).

## Results

Our review identified 433 patients with endometrial serous cancer, 71 with FIGO stage IA disease. In eight patients, adjuvant therapy and outcome data were not available; therefore, 63 patients were included in our analysis. All patients received total hysterectomy and bilateral salpingo-oophorectomy, 50 with surgical staging (79.4%) and 13 without. Patients were considered surgically staged if they had pelvic lymph node dissection and omentectomy during surgery. Some patients treated early in this cohort were not staged, as this was not routinely done at our center until 2005-2006. In those patients who were not surgically staged, six were observed. Median age was 68.4 years (range 45-90) and median follow-up was 38 months (range 3-114). All patients completed chemotherapy except for one who stopped after five cycles and the decision to use single versus double-agent chemotherapy was at the discretion of the treating gynecologic oncologist. In all cases of combined chemotherapy and VBT, a "sandwich" approach was used, with three cycles of chemo given prior to and following VBT. The pathologic and treatment characteristics for the cohort are summarized in Table 1. In the overall cohort, there were 11 recurrences, eight of which were in surgically staged patients, 17.5% and 16% crude recurrence rates, respectively, as summarized in Table 2. RFS was 87.9% at two years and 76.5.% at five years for the cohort, 89.6% at two years, and 79.9% at five years for those with surgical staging only. OS was 94.7% and 84.7% at two and five years, respectively, for the entire cohort, and 97.9% and 90.6% at two and five years for those with surgical staging. The differences in RFS and OS were not statistically significant between the entire cohort and the surgical staging subset.

**Table 1:** Patient pathologic and treatment characteristics VBT = vaginal brachytherapy; EBRT = external beam radiotherapy

	All Patients (N=63)	Surgically Staged (N=50)	No Adjuvant Treatment (N=32)	VBT+Chemotherapy (N=21)
Characteristic	N (%)	N (%)	N (%)	N (%)
PATHOLOGIC CHARACTERISTICS				
Pelvic Nodal Dissection	50 (79.4)	50 (100)	22 (68.8)	21 (100)
Median Pelvic Nodes Removed	9 (range 0-39)	11 (range 2-39)	5 (range 0-39)	12 (range 2-23)
Para-Aortic Nodal Dissection	15 (23.8)	14 (28)	3 (9.4)	11 (52.4)
Omentum Not Removed	39 (61.9)	39 (52)	22 (68.8)	9 (39.1)
Omentum Removed, Not Involved	24 (38.1)	24 (48)	10 (31.2)	12 (60.9)
Myometrium Not Invaded	32 (50.8)	27 (54)	19 (59.4)	10 (47.6)
Myometrium Invaded	31 (49.2)	23 (46)	13 (40.6)	11 (52.4)
Pure Serous histology	58 (92)	46 (92)	28 (87.5)	20 (95.2)
Mixed Serous Histology	5 (8)	4 (8)	4 (12.5)	1 (4.8)
Lymphovascular Invasion Absent	40 (63.5)	36 (62)	18 (56.3)	15 (71.4)
Lymphovascular Invasion Present	3 (4.7)	2 (4)	2 (6.3)	0 (4)
Lymphovascular Invasion - Unknown	20 (31.8)	12 (24)	12 (37.4)	6 (28.6)
TREATMENT CHARACTERISTICS				
Observation	32 (50.8)	22 (44)		
VBT + Chemotherapy (total)	21 (33.3)	21 (42)		
VBT + Chemotherapy (carboplatinum plus paclitaxel)	18 (28.6)	18 (36)		
VBT + Chemotherapy (carboplatinum single agent)	3 (4.7)	3 (6)		
Chemotherapy Alone	7 (11.1)	7 (14)		
VBT Alone	0	0		
Pelvic EBRT + Chemotherapy	1 (1.6)			
Pelvic EBRT Alone	1 (1.6)			

**Table 2:** Characteristics of patients who developed recurrence MI = myometrial invasion; LVI = lymphovascular space invasion; NA= not available; CT = chemotherapy; VBT = vaginal brachytherapy; EBRT = external beam radiotherapy; DOD = died of disease; AWD = alive with disease; AND= alive no disease

	Surgically staged	MI (%)	LVI	Treatment	First recurrence site	Time to recurrence (months)	Salvage therapy	Months alive after recurrence	Alive or dead
1	N	20	Y	OBS	VR, distant	21	None	3	DOD
2	N	25	Y	EBRT	PR, distant	8	None	7	DOD
3	Y	15	NA	OBS	PR, distant	9	Chemo	11	DOD
4	Y	5	NA	OBS	VR, distant	35	Chemo + EBRT+ VBT	11	DOD
5	N	0	NA	OBS	PR, distant	60	Provera	12	AWD
6	Y	3	NA	OBS	VR	11	Chemo + EBRT	65	DOD
7	Y	14	N	VBT + CT	Distant	11	Chemo	53	AWD
8	Y	5	N	VBT + CT	VR	28	Chemo	9	AWD
9	Y	40	NA	VBT + CT	Distant	16	Chemo	11	DOD
10	Y	15	NA	OBS	VR	9	Chemo + EBRT + VBT	32	AND
11	Y	14	NA	VBT + CT	Distant	25	Chemo	8	AWD

Thirty-two patients received no adjuvant therapy and of these, six recurred (18.8% crude recurrence rate). Twenty-one patients received chemotherapy and brachytherapy and four recurred (19.1% crude recurrence rate). The remaining patients received chemotherapy (n=7), EBRT (n=2), or a combination of EBRT and chemotherapy (n=1), and one of the EBRT-only patients faced a recurrence. See Table 3 for areas of first recurrence by treatment type. There were no significant differences in the site of the first recurrence between treatment groups (p=1.0). Five patients who received brachytherapy developed grade 1-2 vaginal stenosis treated with vaginal dilations, and no other radiotherapy-induced complications were reported.

**Table 3:** Areas of first recurrence by treatment type VBT = vaginal brachytherapy; EBRT = external beam chemotherapy

	No adjuvant therapy (Observed)	Chemotherapy and VBT	EBRT alone (n=2)	Chemotherapy alone (n=7)	EBRT plus chemotherapy (n=1)
No recurrence	26	17			
Recurrence in vagina	2	1			
Recurrence in vagina and distant metastasis	2	0			
Recurrence in pelvis and distant metastasis	2	0	1		
Recurrence as distant metastasis	0	3			

The majority of patients received observation or chemotherapy plus VBT, and these groups were therefore compared. The five-year RFS was 73.5% and 74.7% in those observed versus those treated with adjuvant VBT and chemotherapy, respectively (p=0.75). The five-year OS in those observed was 79.6% and in those treated with VBT plus chemotherapy was 95% (p=0.38).

In regards to recurrence and salvage, one patient initially observed developed a vaginal recurrence, received chemotherapy, pelvic radiotherapy, and vaginal vault brachytherapy, and has been without disease for 32 months on close follow-up. None of the other patients were successful salvaged. In the observation group, four patients died of t disease, one patient is alive with no known disease, and one patient is alive on best supportive care. In the VBT plus chemotherapy group, one patient died of disease while three are alive on the best supportive care.

The following risk factors were evaluated in the multivariable analysis: depth of invasion (myometrium versus endometrium only), lymphovascular invasion, dissection of pelvic or para-aortic lymph nodes, omentectomy, percent serous histology, and age at diagnosis. None of these factors were significantly associated with inferior RFS on the multivariable analysis.

## Discussion

A recent study comparing adjuvant therapy regimens among endometrial cancers suggests that serous carcinoma patients are at particularly high risk for local and distant recurrence [16]. This highlights the necessity of ensuring these patients are adequately treated even when presenting with an early stage disease, yet there is currently no consensus on an optimal treatment regimen. According to the National Comprehensive Cancer Network (NCCN) guidelines (2018), observation, chemotherapy, radiotherapy, or combination treatment may all be acceptable strategies for patients with stage IA endometrial cancer [17]. Given the rarity of this disease, most studies are retrospective and involve small numbers of patients, mixed histologies and stages, variable treatment regimens among patients, and few events. This creates a difficulty in interpreting the effectiveness of treatment regimens. It is also desirable to avoid over-treatment of patients with early-stage disease, due to the potential toxicity of adjuvant therapy and the resulting negative impact on quality of life [18].

Our study reports the outcomes of a large cohort of stage IA serous endometrial cancer patients. OS in our study was 84.7% at five years, which is consistent with or slightly better than previously reported for serous endometrial cancers (as low as 65%-85%) [2-5,15]. This is in contrast, however, to patients with stage IA endometrioid uterine cancers, who generally have an excellent five-year OS of approximately 88%, with a disease-specific survival potentially greater than 95% [19].

This emphasizes the poor prognosis of serous endometrial cancers, even at an early stage. Furthermore, although salvage treatment may be successful for early-stage endometrioid cancers that recur locally, with 75% of these patients being eligible for salvage therapy and 85% achieving remission [20], almost all patients in our study who recurred ultimately developed a progressive metastatic disease. Consideration of upfront treatment is, therefore, critical in this group.

Ultimately, the challenge in determining appropriate adjuvant treatment in this early-stage, high-risk group is balancing a reduction in recurrence with treatment-related toxicity and complications. Many studies have reported significant toxicity rates in an attempt to treat stage IA patients aggressively with chemotherapy and radiotherapy, and, despite this, RFS remains at around 85% [18,21-23]. A number of local therapies have been used, including brachytherapy, EBRT, and whole abdominal radiotherapy [24-25]. While some centers have adopted brachytherapy as the local therapy in this setting [26-28], many centers continue to use pelvic radiation as the local component of therapy currently [12-13,22-23] and a standard of care for treatment has not been clearly established.

Historically, at our center, patients received no adjuvant therapy for stage IA serous endometrial cancer, however, more recently, a strategy of combined platinum-based chemotherapy and VBT has been implemented. Despite this predominant use of VBT as the adjuvant radiotherapy, in contrast to EBRT, our rate of local recurrence was consistent with reports at 20%-25% at five years [22]. There may, therefore, be a role for the de-escalation of treatment to the vaginal vault while sparing patients the toxicity of EBRT to the entire pelvis, where toxicity has been reported at 25% [20]. While we observed no statistically significant differences in recurrence between the observation and treatment groups, the literature indicates recurrence rates are quite high in patients receiving no adjuvant therapy [12-13,29]. Furthermore, successful salvage may be difficult in these patients compared with recurrent cases of endometrioid histology and we, therefore, would not recommend omitting adjuvant treatment. We, instead, believe that in surgically staged patients, limiting radiotherapy to vault brachytherapy, along with systemic treatment, may be adequate and minimize morbidity. We were unable to compare the use of VBT with EBRT, however, because few patients received EBRT in our study. Our low vaginal and pelvic recurrence rates with VBT warrant further investigation, as many centers continue to treat early serous cancers with pelvic EBRT [12-13,23].

The rates of distant recurrence in our patients were high (eight of 11 recurrent cases), indicating the systemic recurrence risk in this group. The distant recurrence rates, however, were similar in our patients, regardless of whether systemic therapy was given. Other studies have reported higher stage IA recurrence rates when chemotherapy is omitted, even with radiotherapy, compared to our results [25,28]. For example, Fader et al. report a retrospective study of stage IA patients demonstrating a recurrence of 15.8% with no adjuvant therapy versus 7.4% for those who received chemotherapy and radiotherapy combined [12]. Hamilton et al. found in early stage (I and II) patients, adjuvant radiotherapy and chemotherapy had a significantly improved overall (85% vs. 54%) and disease-free survival (85% vs. 49%) in comparison with observation [13]. Finally, a large retrospective analysis of the National Cancer Database found that chemotherapy was associated with improved survival (22% reduction in mortality) in early-stage serous uterine cancer (IA-II), however, in stage IA patients, specifically, only 34% of patients received chemotherapy and there was no significant mortality benefit (HR 0.88, 0.72-1.08) [30]. Vaginal brachytherapy was interestingly associated with significantly improved survival (HR 0.67, 0.52-0.86) while EBRT is associated with survival detriment (HR 1.26, 1.04-1.54) in stage IA patients. These differences may be explained by a selection bias in serous endometrial cancer studies, with chemotherapy or radiotherapy offered to higher-risk patients. The variability in these findings indicates larger controlled studies are required to evaluate the degree of effectiveness of adjuvant chemotherapy combined with radiotherapy in this population. Nevertheless, given the high systemic risk, the authors of our study still advocate for the use of chemotherapy in an attempt to improve outcomes.

Of the pathologic factors, patient factors, and treatment factors analyzed in our study, there were no significant associations with recurrence. Other studies have strongly suggested myometrial invasion as a risk factor [12,29]; however, an endometrium-confined disease must not be underestimated, as recurrence rates may still be high without adjuvant therapy in these patients [23]. Peritoneal spread, lymph node involvement, lack of surgical staging, and lymphovascular invasion have also been identified as significant recurrence risks and require further characterization [5]. Likewise, surgical staging has been identified as a prognostic factor for this disease [16], however, we found no difference in outcomes in those with surgical staging. This emphasizes the lack of predictability of this disease in patterns of recurrence, warranting careful consideration of adjuvant treatment.

Interestingly, overall, only one patient with an endometrium-confined disease (and no adjuvant treatment) recurred. This represents only a 3% recurrence in endometrium-confined patients and 5% in patients with no adjuvant therapy. This suggests that a de-escalation of treatment in these patients could be considered and further investigation is warranted.

This study reports one of the largest cohorts of stage IA serous endometrial cancer patients to date. The limitations of our study include the small sample size, with few events, despite having a relatively large cohort of stage IA patients for this rare disease. The retrospective nature prevents the opportunity to standardize treatment regimens and excludes a bias in decision-making for observation versus combined adjuvant therapy.

The strength of this study is the focus on only serous histology and early stage IA patients only; a group where there is significant uncertainty regarding the benefit of adjuvant therapy. In addition, a sizable number of patients received no adjuvant therapy after surgery, which gives us an opportunity to document the patterns of relapse in stage IA patients. In those who did receive adjuvant chemotherapy and brachytherapy treatment, an excellent five-year survival of 95% was observed. In comparison, the group with no adjuvant treatment had a five-year OS of 79.6% though the difference was non-significant (p=0.38). Additionally, recurrence is generally associated with poor outcomes, as was observed in our study. Clinicians may, therefore, use our results to understand the behavior of this disease in both early-stage groups.

Larger, multicenter, randomized studies are required to determine the optimal adjuvant therapy regimen for patients with stage IA serous endometrial cancers and to further characterize risk factors for recurrence and progression. In the meantime, based on our findings, we suggest that chemotherapy remains a critical component of treatment given the high rates of distant recurrence while brachytherapy appears sufficient for minimizing local recurrence without the requirement of pelvic radiotherapy in serous stage IA patients.

## Conclusions

As anticipated in our cohort of early stage serous endometrial cancer patients, RFS and OS were inferior to those reported for patients with endometrioid histology and recurrence is associated with poor outcomes. This supports a consideration of adjuvant therapy even in very early-stage disease. While pelvic radiotherapy is still used in many centers, and there is no universal standard of care currently, our results support vaginal brachytherapy as an adequate local treatment. Although firm conclusions regarding optimal adjuvant therapy cannot be made given the small numbers, variability in adjuvant treatment, and the potential selection bias given the retrospective study design, the regimen of combined brachytherapy and chemotherapy appears to be promising and warrants further investigation.
